# Factors associated with hospital revisitation within 7 days among patients discharged at triage: a case–control study

**DOI:** 10.1097/MEJ.0000000000001156

**Published:** 2024-07-04

**Authors:** Jari Ylä-Mattila, Teemu Koivistoinen, Henna Siippainen, Heini Huhtala, Sami Mustajoki

**Affiliations:** a Emergency Department, Tampere University Hospital; b Faculty of Medicine and Health Technology, Tampere University, Tampere; c Emergency Department, Kanta-Häme Central Hospital, Hämeenlinna; d Faculty of Social Sciences, Tampere University, Tampere, Finland

**Keywords:** discharge, emergency department, low acuity, nonurgent patient, redirect, triage

## Abstract

**Background and importance:**

Existing data are limited for determining the medical conditions best suited for an emergency department (ED) redirection strategy in a heterogeneous, nonurgent patient population.

**Objective:**

The aim was to establish factors associated with hospital revisits within 7 days among patients discharged or redirected by a triage team.

**Design, settings, and participants:**

An observational single-center case–control study was conducted at the Tampere University Hospital ED for the full calendar year of 2019. The cases comprised unplanned hospital revisits within 7 days of being discharged or redirected by triage, while the controls were discharged or redirected but did not revisit.

**Outcome measures and analysis:**

The primary outcome was an unplanned hospital revisit within 7 days. A subgroup analysis was conducted for revisits leading to hospitalization. Basic demographics, comorbidities before triage, and triage visit characteristics were considered as predictive factors for the revisit. A backward stepwise conditional logistic regression analysis was performed.

**Main Results:**

During the calendar year of 2019, there were a total of 92 406 ED visits. Of these, 7216 (7.8%) visits were discharged or redirected by triage, and 6.5% (*n* = 467) of all these patients revisited. Of the revisiting patients, 25% (*n* = 117) were hospitalized. In multivariable analysis, higher age was associated with both revisitation [odds ratio (OR): 1.01, 95% confidence interval (CI): 1.00–1.02] and hospitalization (OR: 1.02, 95% CI: 1.00–1.04). Furthermore, using other visits as a reference, abdominal pain was associated with revisitation and hospitalization (OR: 3.70, 95% CI: 2.24–6.11 and OR: 5.28, 95% CI: 2.08–13.4, respectively).

**Conclusion:**

Higher age and abdominal pain were associated with hospital revisitation and hospitalization within 7 days among patients directly discharged or redirected by the triage team. Regardless of the triage system in use, there might be patient groups that should be evaluated more cautiously if a triage-based discharge or redirection strategy is to be considered.

## Introduction

Increased demand for emergency department (ED) services is a worldwide phenomenon [1]. Because of the heterogeneity of ED patients, the importance of more accurate and appropriate triage has increased, regardless of the triage system [2]. The proportion of ED visits classified as nonurgent and the definitions of such visits vary greatly [3,4].

The principal purpose of ED triage is to ensure that patients receive the level and quality of care appropriate to their clinical needs and that departmental resources are most effectively applied to this end [5]. The ability of triage, however, to predict hospitalization is considered suboptimal [6–8]. Furthermore, attempts to produce a predictive model for refusal of nonurgent ED care have not succeeded [9–13], and most nonurgent visits eventually require some interventions at the ED [14]. Some evidence suggests that ED redirection is safe [11,12,15–18], but the data are limited with regard to concluding which medical conditions are suitable for ED redirection [12] in a heterogeneous nonurgent patient population [19]. An earlier study showed that 92% of patients discharged by triage did not require subsequent ED admission within 7 days, and the 30-day mortality rate was 0.07% for discharged patients [20].

Knowledge about the factors associated with the hospital revisits of patients redirected by ED triage has been limited [12]. To enhance future triage performance and accuracy, the factors affecting the later hospital revisitation of discharged or redirected patients should be described in more detail. The purpose of this case–control study was to determine factors associated with (1) unplanned hospital revisits within 7 days and with (2) revisits within 7 days that lead to hospitalization among a group of patients initially directly discharged or redirected by the triage team.

## Methods

### Study design and setting

A single-center observational case–control study was conducted at the ED of Tampere University Hospital, Finland. Chart review data were collected retrospectively from 1 January to 31 December 2019, from the hospital’s electronic patient records. The study was approved by the hospital’s research director (research diary number R21511). Finnish law does not require ethics committee approval for register studies [21]. The STROBE (Strengthening the Reporting of Observational Studies in Epidemiology) guidelines were applied in this study [22].

Tampere University Hospital provides secondary care for over 500 000 residents in the Pirkanmaa Hospital District (universal publicly funded healthcare system) and is the only hospital in the region managing all severe emergency situations. In addition, the hospital is a tertiary care unit for a catchment area of over 900 000 residents. The ED serves only adult patients, with the exception of injuries in patients under 16 years of age. All patients entering the ED are evaluated by the triage team, and the aim is to directly discharge or redirect low-acuity patients to nonurgent healthcare services (e.g. healthcare centers, occupational or private healthcare). Outside normal working hours, only a few other healthcare providers are available in the region, which increases the proportion of lower acuity patients in the ED.

A five-level triage system, the Emergency Severity Index (ESI) [23], is used in the ED. The triage team is composed of trained nurses and a physician is always available for consultation either by telephone or, if necessary, being present in the triage situation. Triage nurses assess the ESI class independently, and they are also allowed to directly discharge or redirect patients to other suitable healthcare providers outside the ED without consulting a physician, but they are encouraged to consult a physician in any undetermined cases. One nurse is responsible for triaging patients arriving by ambulance, and two to five nurses perform triage on walk-in patients. The clinical urgency evaluation consists of the patient’s medical history, an interview, an evaluation of the severity of symptoms, and, if appropriate, the measurement of vital signs and point-of-care analyses, including C-reactive protein. The final decision, however, to discharge or redirect a patient is based on clinical decision-making with or without a physician. The ESI classification [23] or the National Early Warning Score [24], a tool for the detection of and response to clinical deterioration in adult patients, is not used for directly discharged or redirected patients.

### Study protocol and population

**Fig. 1:**
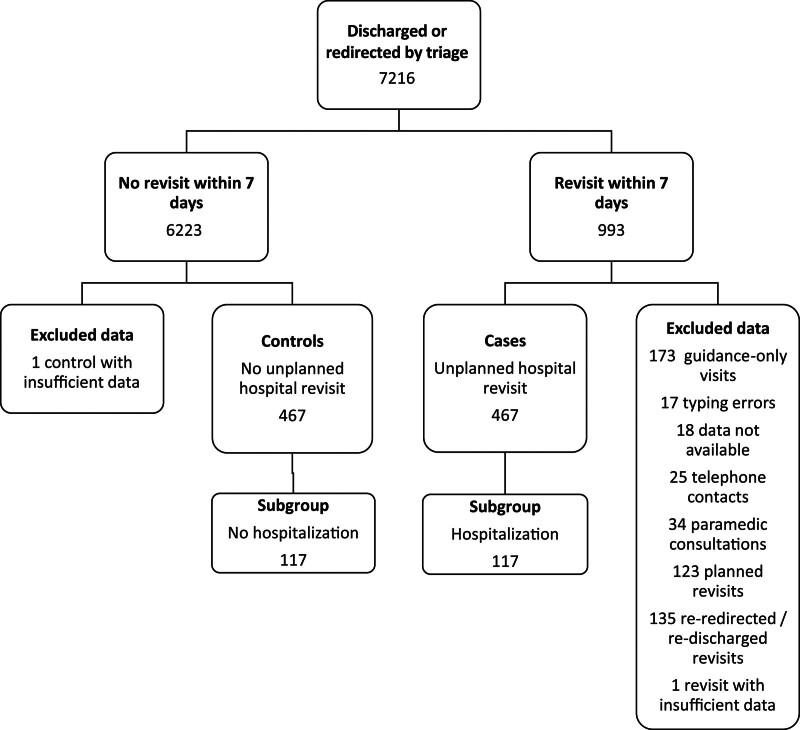
Study selection flowchart.

The study population consisted of patients who were evaluated and discharged or redirected by the ED’s triage team. The hospital’s data management services provided the list of these patients, with time stamps and admissions data. A case–control study setting was devised to explain factors associated with revisits and hospitalization. The cases comprised unplanned revisits by patients who had completed the registration process and been admitted to the ED or other hospital units (pediatric ED, obstetrics, and gynecology emergency unit, a regional hospital ED, or the cardiac care unit) within 7 days after discharge or redirection. The control patients were also discharged or redirected by triage but did not revisit within 7 days. The subgroup analysis entailed hospitalized patients, including one who discharged himself against medical advice from the ED. Excluded cases consisted of visiting patients who were guided to the appropriate unit within the hospital district, typing errors, cases with data not available, telephone contacts, or paramedic consultations. Furthermore, planned revisits and re-redirected or re-discharged revisits were excluded. One case–control pair was excluded because of missing data from the control patient (Fig. 1).

The case–control pairs were collected in a 1 : 1 ratio, and the discharged or redirected control patient for each case was the one with the time stamp closest to that of the index patient. Using time as a criterion aimed at controlling for confounding factors independent of the patient, such as triage shifts, the time of day and season, ED and hospital crowding, and the availability of other healthcare providers. Basic demographics, comorbidities before the ED triage visit, and triage visit characteristics were examined as predictive factors for the hospital revisit.

### Study variables

The collected explanatory variables of each visit are presented in detail in Supplementary File I, Supplemental digital content, *http://links.lww.com/EJEM/A442*. The updated Charlson Comorbidity Index (uCCI) [25], with *International Classification of Diseases 10*^*th*^ *Revision* coding algorithms [26], was used. National Early Warning Score [24] values were not calculated routinely for every patient redirected by triage; for this study, the retrospectively reconstructed values (reconstructed National Early Warning Score) were created using the data of measured physiological parameters, if measured. In addition, the numeric C-reactive protein value was recorded, if measured.

### Data analysis

Continuous data are presented as medians and interquartile ranges (IQR), whereas categorical data are given as numbers and percentages. The predictors for matched case–control pairs were assessed with univariate and multivariable logistic regression using backward conditional stepwise selection with a significance level of 0.1. Analyses were performed, and the results were presented as odds ratios (ORs) with 95% confidence intervals (CIs). *P*-values below 0.05 were considered statistically significant.

In the conditional logistic regression analyses, age and the uCCI were regarded as continuous variables. The categorical variables were sex, comorbidities, and physician consultation. Furthermore, as regards the categorical variables of the main complaints, ‘other’ was regarded as a reference because it was the most abundant group. In addition, physiological parameters or C-reactive protein were regarded as unnecessary in the triage process, if they were not assessed. The continuous data of the National Early Warning Score and C-reactive protein values were classified into three categories, and in both variables, ‘not measured’ was considered as a reference. Statistical analyses were performed with Stata (v.17.0; StataCorp, College Station, Texas, USA).

## Results

### Study population

During the calendar year of 2019, there were a total of 92 406 ED visits. Of these, 7216 (7.8%) were discharged or redirected by triage, and 6.5% (*n* = 467) of all these patients had an unplanned hospital revisit within 7 days. Of these revisiting patients, 117 (25%) were hospitalized (Fig. 1). The proportion of the male patients was somewhat similar among the revisit (*n* = 212) and no revisit (*n* = 213) groups: 45.4 and 45.6%, respectively. The median age was higher in the revisit group than in the no revisit group, 38 (IQR: 26–57) and 32 (IQR: 22–56) years, respectively (Table 1).

**Table 1:** Factors predicting hospital revisits within 7 days (*N* = 934)

	Characteristics		Conditional logistic regression					
	Revisit (*N* = 467)	No revisit (*N* = 467)	Univariate			Multivariable^a^		
	*n* (%)/median (IQR)	*n* (%)/median (IQR)	OR	95% CI	*P*-value	OR	95% CI	*P*-value
Basic demographics								
Male sex	212 (45.4)	213 (45.6)	0.99	0.77–1.28				
Age, years	38 (26–57)	32 (22–56)	1.01	1.00–1.02	0.003	1.01	1.00–1.02	0.008
Comorbidity								
Updated Charlson Comorbidity Index	0 (0–0)	0 (0–0)	1.17	1.01–1.35	0.035			
uCCI score = 0	376 (80.5)	392 (83.9)						
uCCI score = 1–3	77 (16.5)	72 (15.4)						
uCCI score > 3	14 (3.0)	3 (0.6)						
Other comorbidities								
Mental health disorder	113 (24.2)	52 (11.1)	2.45	1.71–3.51	<0.001	2.30	1.55–3.41	<0.001
Substance use disorder	78 (16.7)	23 (4.9)	2.57	1.55–4.26	<0.001			
ED triage visit characteristics								
Main complaint								
Other	325 (69.6)	417 (89.3)	Reference			Reference		
Abdominal pain	71 (15.2)	30 (6.4)	3.26	2.01–5.29	<0.001	3.70	2.24–6.11	<0.001
Mental health or substance abuse problem	38 (8.1)	7 (1.5)	6.95	3.03–15.9	<0.001	5.10	2.15–12.1	<0.001
Neurological or vision	33 (7.1)	13 (2.8)	3.01	1.56–5.83	0.001	2.90	1.46–5.74	0.002
Physiological parameters								
Not measured	265 (56.8)	296 (63.4)	Reference					
Measured, total rNEWS = 0	137 (29.3)	112 (24.0)	1.34	1.00–1.80	0.046			
Measured, total rNEWS ≥ 1	65 (13.9)	59 (12.6)	1.25	0.83–1.87				
Point-of-care analyses								
C-reactive protein								
Not measured	370 (79.2)	406 (86.9)	Reference					
≦10 mg/L	65 (13.9)	43 (9.2)	1.67	1.11–2.50	0.014			
>10 mg/L	32 (6.9)	18 (3.9)	2.13	1.13–4.01	0.019			
Physician consulted	188 (40.3)	168 (36.0)	1.23	0.93–1.64				

CI, confidence interval; IQR, interquartile range; OR, odds ratio; rNEWS, reconstructed National Early Warning Score; uCCI, updated Charlson Comorbidity Index (maximum comorbidity score 24) with *International Classification of Diseases, 10*^*th*^ *Revision* coding algorithms [25,26].

a Backward stepwise, pseudo *R*^2^ = 0.14.

### Hospital revisits

In the multivariable analysis, the variables associated with revisitation were higher age, with an OR for a 1-year increase of 1.01 (95% CI: 1.00–1.02), and a prior mental health disorder (OR: 2.30, 95% CI: 1.55–3.41). Furthermore, using other visits as a reference, abdominal pain (OR: 3.70, 95% CI: 2.24–6.11), neurological or vision symptoms (OR: 2.90, 95% CI: 1.46–5.74), and mental health or substance abuse problem (OR: 5.10, 95% CI: 2.15–12.1) were associated with a higher revisitation probability. A physician was consulted more often in the case group than in the control group, with consultation rates of 40.3 and 36.0%, respectively, but the difference was statistically NS. The most common uCCI score in both the case and the control group was 0 (IQR: 0–0), for 81 and 84% of the patients, respectively. Furthermore, the highest scores in the two groups were 7 and 6, respectively, while the maximum score was 24. Higher scores, however, were not associated with revisits (Table 1).

### Revisits leading to hospitalization

In the multivariable analysis of this subgroup, the variables associated with hospitalization were higher age, with an OR: for a 1-year increase of 1.02 (95% CI: 1.00–1.04), and the higher uCCI score (OR: 1.45, 95% CI: 1.01–2.09). In both, the case and the control groups, the maximum score was 6. In addition, abdominal pain patients (OR: 5.28, 95% CI: 2.08–13.4) and patients with mental health or substance abuse problem (OR: 17.1, 95% CI: 1.99–148) were more likely to be hospitalized than was the reference group of patients with other complaints. Furthermore, a reconstructed National Early Warning Score of 1 or more was associated with hospitalization (OR: 2.72, 95% CI: 1.09–6.83). A physician, however, was consulted in 39.3% of the cases, which did not differ from the control group (38.5%) (Table 2).

**Table 2:** Factors predicting hospitalization within 7 days (*N* = 234)

	Characteristics		Conditional logistic regression					
	Hospitalization (*N* = 117)	No hospitalization (*N* = 117)	Univariate			Multivariable^a^		
	*n* (%)/median (IQR)	*n* (%)/median (IQR)	OR	95% CI	*P*-value	OR	95% CI	*P*-value
Basic demographics								
Male sex	59 (50.4)	55 (47.0)	1.13	0.69–1.85				
Age, years	43 (32–68)	32 (20–59)	1.02	1.01–1.03	0.001	1.02	1.00–1.04	0.013
Comorbidity								
Updated Charlson Comorbidity Index	0 (0–1)	0 (0–0)	1.50	1.12–2.00	0.006	1.45	1.01–2.09	0.045
uCCI score = 0	79 (67.5)	95 (81.2)						
uCCI score = 1–3	29 (24.8)	21 (17.9)						
uCCI score > 3	9 (7.7)	1 (0.9)						
Other comorbidities								
Mental health disorder	19 (16.2)	17 (14.5)	1.13	0.57–2.27				
Substance use disorder	19 (16.2)	6 (5.1)	3.17	1.26–7.93	0.014			
ED triage visit characteristics								
Main complaint								
Other	67 (57.3)	104 (88.9)	Reference			Reference		
Abdominal pain	30 (25.6)	9 (7.7)	4.65	1.99–10.8	<0.001	5.28	2.08–13.4	<0.001
Mental health or substance abuse problem	13 (11.1)	1 (0.9)	18.9	2.31–154	0.006	17.1	1.99–148	0.010
Neurological or vision	7 (6.0)	3 (2.6)	2.79	0.69–11.3				
Physiological parameters								
Not measured	56 (47.9)	75 (64.1)	Reference			Reference		
Measured, total rNEWS = 0	33 (28.2)	31 (26.5)	1.41	0.77–2.59	0.264			
Measured, total rNEWS ≥ 1	28 (23.9)	11 (9.4)	3.24	1.47–7.13	0.003	2.72	1.09–6.83	0.033
Point-of-care analyses								
C-reactive protein								
Not measured	86 (73.5)	101 (86.3)	Reference					
≦10 mg/L	18 (15.4)	12 (10.3)	1.75	0.80–3.83				
>10 mg/L	13 (11.1)	4 (3.4)	6.08	1.33–27.8	0.020			
Physician consulted	46 (39.3)	45 (38.5)	1.05	0.58–1.88				

CI, confidence interval; IQR, interquartile range; OR, odds ratio; rNEWS, reconstructed National Early Warning Score; uCCI, updated Charlson Comorbidity Index (maximum comorbidity score 24) with *International Classification of Diseases, 10*^*th*^ *Revision* coding algorithms [25,26].

a Backward stepwise, pseudo *R*^2^ = 0.31.

## Discussion

The present research used a case–control study design to examine factors associated with an unplanned hospital revisit and hospitalization within 7 days of an initial triage discharge or redirection in a large Finnish tertiary care hospital. Overall, 7.8% of visits were discharged or redirected, and 6.5% of all these patients revisited the hospital within 7 days. Of the revisiting patients, 25% were hospitalized.

Knowledge about the factors associated with hospital revisits of patients redirected by ED triage has been limited [12]. The present results demonstrate that higher age was associated with hospital revisitation and hospitalization. In line with previous studies, age over 65 years has been associated with hospitalization in a nonurgent patient population [14,27,28]. Furthermore, abdominal pain as a main complaint was significantly associated with hospital revisitation and subsequent hospitalization within 7 days. In a prior study, 22% of patients who reported abdominal pain were hospitalized nonelectively within 1 week [11]. It was also the most reported symptom in urgent and semiurgent patient groups [19] and was judged to be less than safe for deferred care [11]. Some medical conditions may become more evident as they progress with time, which possibly explains why patients with abdominal pain are not evaluated as flawlessly as other patients. A previous study, however, in our ED showed that 3% of patients discharged with a diagnosis of nonspecific abdominal pain revisited the ED within 48 h and that 0.7% were hospitalized despite the ED admission during the first visit [29].

Patients with an altered mental state have previously been systematically excluded because of a lack of criteria and categorization consensus [4]. In the present study, a prior mental health disorder with continuous medication was associated with revisitation, and mental health or substance abuse problem with revisitation and hospitalization. The role of the ED as a 24/7 safety net provider for the most vulnerable members of society has been recognized for decades [10,30]. Some of the revisits might be explained by organizational reasons having to do with the healthcare system (e.g. access to a primary care provider) [19], or by the patient visiting the ED out of habit. However, only a small number of revisiting patients with acute mental health or substance abuse problems were included in the subgroup analysis of hospitalized patients, which indicates that the triage team’s ability to recognize the need for specific acute hospital care was sufficient in these patient groups.

Triage nurses’ ability to predict or categorize the extremes of triage urgency is accurate but varies between diagnostic groups when predicting ED discharge [7], and their ability to predict hospitalization is thus considered suboptimal [6–8]. The present study, however, also showed variation between diagnostic groups, for example, neurological or vision symptoms were associated only with revisits but not with hospitalizations. Furthermore, the percentage of physician consultations was equal in the subgroup of hospitalized patients in both the case and the control group, although this finding might be biased by the fact that a physician was consulted only when a patient’s symptoms were somehow ambiguous. However, similarly to a prior study in which Afilalo *et al*. [19] reported that ED patients classified as ‘nonurgent’ have fewer prior medical conditions, the median uCCI was 0 in both the revisit group and in the subgroup of hospitalized patients who were initially triaged as ‘nonurgent’ and discharged. Nevertheless, the proportion of uCCI scores was higher in both the revisit and the hospitalization group, and the higher scores were associated with hospitalization in multivariable analyses. Likewise, deviations in physiological parameters were associated with hospitalization. However, to enhance future triage performance, the most practical means would be to recognize medical conditions unsuitable for ED-based redirection or direct discharge from triage.

### Limitations

The present study also has its limitations. This was a single-center retrospective case–control chart review study covering a period of 1 year. While the data on subsequent ED and hospital utilization are comprehensive, the eventual ambulatory utilization of healthcare services remains unknown because there was no regular follow-up for discharged and redirected patients or because triage could not specifically redirect patients to a primary care provider. Therefore, it is possible that revisits may have taken place at primary healthcare units or hospitals located outside the Pirkanmaa Hospital District. Tampere University Hospital, however, is the only hospital in the region managing all severe emergency situations. The distance to the closest hospital with similar resources is 80 km, which justifies the assumption that our data include at least the vast majority of revisits requiring a secondary care hospital service.

The current study was also susceptible to chart review study biases. The chief complaints of patients were collected retrospectively from electronic health records. There was no validated or structured practice for documentation, and the data had therefore not been collected systematically at triage. This may have influenced the recording of the most urgent complaint, especially if a patient had multiple complaints. Furthermore, the results may be somewhat biased because the reason for the revisit may differ from the first visit [20]. It is also possible that, even if vital signs were measured, point-of-care analyses performed, or a physician consulted during the triage visit, these were not recorded in the electronic health records. In addition, basic demographic variables were limited only to age and sex. Other demographic factors potentially related to nonurgent ED utilization were not collected, nor were the convenience of ED care or access to primary care [3,19].

The case–control-matched pairs were collected at a 1 : 1 ratio. The number of patients was limited, particularly in the subgroup analysis of unplanned visits leading to hospitalization. However, the controls were collected from the same population, and they were the closest in time to their case pairings. Thus, the confounding factors were considered. The relatively low number of patients, however, might limit the statistical power to demonstrate some clinically relevant associations in analyses, even if there were some.

## Conclusion

According to this study, higher age was associated with hospital revisitation and hospitalization within 7 days among patients discharged or redirected by the triage team. Furthermore, patients with abdominal pain were more likely to revisit and be hospitalized than patients with other complaints. Regardless of the triage system in use, there might be patient groups that should be evaluated more cautiously if a triage-based discharge or redirection strategy is to be considered.

## Acknowledgements

This research was funded by the Research Services of the Pirkanmaa Hospital District, grant number MJ0067.

### Conflicts of interest

There are no conflicts of interest.

## Supplementary Material


