# Supplementary material for: Factors associated with hospital revisitation within 7 days among patients discharged at triage: a case–control study
Source: Eur J Emerg Med. 2024 Jul 4;32(1):22–8. doi: 10.1097/MEJ.0000000000001156 (PMC11665969; doi:10.1097/MEJ.0000000000001156)
Supplement: Supplementary file 1 [file ejem-32-22-s001.pdf]

Supplementary file I. Predictive variables

	Variables
**Basic demographics**	Personal identity code, sex, date of birth, and age upon arrival.
**Comorbidities prior to visit**	Updated Charlson Comorbidity Index, mental health disorder with continuous medication, substance use disorder.
**ED triage visit characteristics**	
Category of the main complaint	Other: (minor trauma, infection [cough, fever, sore throat, urinary tract infection], non-traumatic pain [back, upper or lower extremities, tooth], cardio-respiratory [dyspnea, chest pain presumably of non-cardiac origin, palpitation], nausea/emesis, non-traumatic bleeding [gynecological, epistaxis, hematochezia], and miscellaneous complaints, including multiple complaints).<br>Abdominal pain, mental health or substance abuse problem, neurological (headache, dizziness, passed disorientation) or vision symptoms.
Physiological parameters	Numeric values of heart rate (beats per minute), systolic blood pressure (millimeters of mercury), pO_2 saturation (oxygen saturation %), respiration rate (breaths per minute), body temperature (degrees centigrade), and level of consciousness.

Point-of-care analysis	Numeric value of C-reactive protein.
Consultations	Whether physician was consulted either by telephone or with the physician present.

Updated Charlson Comorbidity Index (maximum comorbidity score 24) with ICD-10 coding algorithms [25,26].
